# Systematic review of public health research on prevention of mother-to-child transmission of HIV in India with focus on provision and utilization of cascade of PMTCT services

**DOI:** 10.1186/1471-2458-12-320

**Published:** 2012-05-02

**Authors:** Shrinivas Darak, Mayuri Panditrao, Ritu Parchure, Vinay Kulkarni, Sanjeevani Kulkarni, Fanny Janssen

**Affiliations:** 1 Population Research Centre, Faculty of Spatial Sciences, University of Groningen, Groningen, The Netherlands; 2 PRAYAS Health Group, Pune, India; 3 Department of Epidemiology, University of California Berkeley, Berkeley, CA, USA; 4 Department of Epidemiology, University of South Florida, Tampa, FL, USA

**Keywords:** PMTCT, India, Systematic review, Research output

## Abstract

**Background:**

In spite of effective strategies to eliminate mother-to-child-transmission of HIV, the implementation of such strategies remains a major challenge in developing countries. In India, programs for the prevention of mother-to-child transmission (PMTCT) have been scaled up widely since 2005. However, these programs reach only a small percentage of pregnant women, and their overall effectiveness is low. Evidence-based program planning and implementation could significantly improve their effectiveness. This study sought to systematically retrieve, thematically categorize and review published research on PMTCT of HIV in India, focusing on research related to the provision and/or utilization of the cascade of services provided in a PMTCT program, in order to direct further research to enhance program implementation and effectiveness.

**Methods:**

A systematic search using MEDLINE, US National Library of Medicine Gateway system (PubMed) and ISI Web of Knowledge resulted in 1,944 abstracts, of which 167 met our inclusion criteria.

**Results:**

A huge share of the empirical literature on PMTCT in India (N = 134) deals with epidemiological studies (N = 60). The 46 papers related to utilization/provision of the cascade of PMTCT services were mostly from the four high HIV prevalence states in southern India and from the public sector. Studies on experiences of implementing a PMTCT program (N = 20) show high rates of drop out of women in the cascade particularly prior to receiving ARV. Studies on individual components of the cascade (N = 26) show that HIV counseling and testing is acceptable and feasible. Literature on other components of the cascade - such as pregnant women’s access to ANC care, HIV infected women’s immunological assessment using CD4 testing, repeat HIV testing among pregnant women, early infant diagnosis and factors related to linking HIV infected women and children to postnatal care – is lacking.

**Conclusions:**

While the scale of the Indian PMTCT program is large, comprehensive understanding of the context-driven factors affecting its efficiency is lacking. Systematic and more focused public health research output is needed on the issues related to reduction of drop outs of women in the cascade, role of PMTCT programs in improving maternal and child health indicators and role of private sector in delivering PMTCT services.

## Background

It has been more than 16 years since a breakthrough clinical trial (ACTG 076) demonstrated that the administration of prophylactic antiretroviral medicine - Zidovudine - to HIV-infected mothers and infants can reduce mother-to-child transmission (MTCT) of HIV by almost 68 percent [1]. Significant advances have been made since then in developing the science of prevention of mother-to-child transmission of HIV (PMTCT). A number of clinical trials have been conducted to evaluate the effectiveness of different antiretroviral drug regimens in different combinations administered for varied durations. As a result, MTCT can now be reduced to less than 2 percent from a possible 25–30 percent without any intervention [2].

With the use of effective antiretroviral treatment (ART) and non-antiretroviral (ARV) strategies, MTCT has been virtually eliminated in developed countries. However, translating the scientific knowledge on an effective ARV regimen and the prevention of transmission through breast milk into field-level action has remained a major challenge for developing countries. Globally, an estimated 3,70,000 children were newly infected with HIV in 2009, and most of them were from developing countries [3]. Further, in 2009, of the women in low- and middle-income countries eligible to receive antiretroviral medication to prevent the mother-to-child transmission of HIV only on average 53% (40% and 79% respectively) received them [3]. In June 2011, a new global plan to eliminate HIV infections among children was launched at the UN [4].

In India, the National AIDS Control Organization (NACO) has adopted the PMTCT program as an important component of its response to the challenge of controlling and reversing the HIV epidemic. The program which is called the PPTCT program (Prevention of Parent-to-Child Transmission) started in 2002, and has been rapidly scaled up in the country through an increase in HIV counseling and testing facilities for pregnant women. The number of integrated counseling and testing centers rose from 2,815 in 2005–6 to 5,135 in 2009–10 [5]. Despite this scale-up in the program, only 20% of the annual estimates of pregnant women were counseled and tested for HIV in 2009. Furthermore, only 30 percent of the estimated HIV infected pregnant women were identified and only 60 percent of those identified as HIV-infected received a single dose of Nevirapine for PPTCT, which was the then National protocol [5].

There are several lacunae in the national PPTCT program. The overall coverage of the cascade of services provided in the program - beginning with counseling and HIV testing for pregnant women followed by immunological assessment of women by CD4 testing, provision of antiretroviral treatment or prophylaxis for the HIV-infected pregnant women, safe obstetric interventions and counseling, support for safer infant feeding options [6] and linking of HIV infected mother and children to postnatal care - has been quite low [7]. In spite of the scientific evidence and recommendations on more effective protocols drawn up by WHO in 2006 [8], and then in 2010 on PMTCT [9], the acceptance of these protocols in the national program has been slow and delayed.

In order to improve the effectiveness of India’s PPTCT program and to meet the goal of achieving the virtual elimination of pediatric HIV in the country, it is important to devise appropriate evidence-based strategies. It is therefore essential to review the existing published literature on the public health aspect of PMTCT in India, in order to facilitate further research and evidence-based planning.

In this paper we present the findings of a systematic review of literature on PMTCT of HIV in India. The objectives of this paper are twofold: first, to thematically categorize the existing peer-reviewed literature on PMTCT in India, and second, to describe the findings of public health literature on PMTCT. For the purpose of this study, public health literature is defined as literature that deals with the provision and/or utilization of the cascade of services provided under a PMTCT project. The findings of this review will help direct future research that can aid program implementation and improve effectiveness of the PMTCT program in India.

## Methods

### Eligibility criteria

For our review of literature on PMTCT of HIV in India, studies were considered relevant if 1) the study was related to HIV and the study population comprised of pregnant women or HIV-exposed children, or 2) the stated objective of the research was directly linked to provision and/or utilization of services provided in a PMTCT project, such as counseling and HIV testing, ARV, obstetric care, infant feeding and infant testing, or 3) the stated objective of the research was directly linked to the planning or provision of PMTCT services, and 4) the study was conducted in India.

### Information sources

We searched MEDLINE and the US National Library of Medicine Gateway system (PubMed) to identify relevant English-language published literature. We also searched reference lists of relevant studies and carried out a cited reference search in ISI Web of Knowledge. We did not include conference proceedings or abstracts when additional information was not provided.

### Search strategy

We used a comprehensive search strategy to identify all relevant research related to the prevention of mother-to-child transmission of HIV in India. Our search strategy included two search terms including the following keywords: 1) (Mother to child transmission, OR MTCT, OR prevention of mother-to-child transmission, OR PMTCT, OR prevention of parent-to-child transmission, OR PPTCT, OR infant feeding, OR HIV infected pregnant women, OR voluntary counseling and testing, OR VCTC, OR integrated counseling and testing, OR ICTC AND India) and 2) (HIV AND pregnancy AND India) We conducted our search on 25 July 2011, and did not include any restrictions on when the research was conducted.

### Data collection process

We exported search results using the reference management software EndNote X4. To determine the relevance of the studies, the first three authors independently reviewed them using a data extraction form to assess the eligibility of studies based on the criteria specified above. Disagreements were resolved by discussion and consensus among all the authors. Of the retrieved papers, details (e.g. abstract, contact information) were not available for two studies [10,11], and hence their relevance could not be assessed. We did not exclude any study based on the methodological quality of the research.

## Results

### Study selection

**Figure 1:**
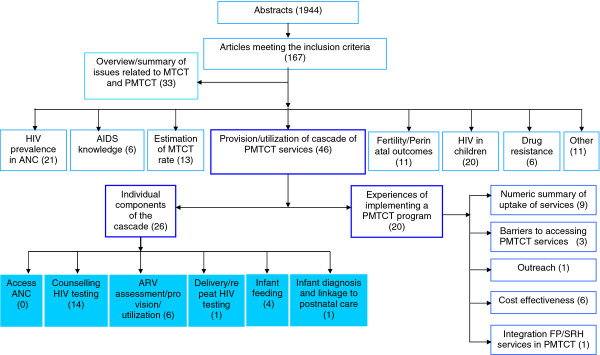
Categorization of published and peer-reviewed literature on PMTCT in India.

We retrieved 1,162 abstracts from the first search term and 238 abstracts from the second term in PubMed and MEDLINE and, in addition, respectively 404 and 140 abstracts through ISI Web of Knowledge, totaling 1,944 abstracts. There were 155 papers from PubMed and an additional 12 papers from ISI Web of Knowledge that met the abovementioned criteria for inclusion. These 167 relevant papers were thematically categorized (Figure 1), and papers related to the provision and/or utilization of one or more services in the PMTCT cascade were identified for an in-depth review (N = 46).

### Thematic categorization of PMTCT literature

The categorization of 167 papers meeting the inclusion criteria is shown in Figure 1. Of these, 33 summarized the existing known theory of mother-to-child transmission, and the interventions to prevent it (for example [12,13]), and this included one book chapter on breast-feeding practices in relation to HIV [14] and a perspective paper on HIV testing in the labor room [15]. The remaining 134 papers involved empirical work. These papers were further classified, based on their primary objective or the main results of the research.

A very large proportion of the empirical literature (N = 134) on PMTCT in India deals with epidemiological studies (N = 60) (45%), including studies that reported the estimated HIV prevalence among pregnant women (N = 21), the observed rate of mother-to-child transmission of HIV in children (N = 13), HIV infection in children (N = 20), and drug resistance among women or children (N = 6). The primary objective or the main results of 46 papers (34%) were related to the provision and/or utilization of the cascade of PMTCT services. In addition, empirical work was done on knowledge about HIV/AIDS among pregnant women (N = 6), fertility desires and perinatal outcomes among HIV-infected women (N = 11), and other subjects (N = 11).

Among the 46 studies related to the provision and/or utilization of services, e.g. the public health literature on PMTCT in India, 20 papers reported on program experiences and were not related to any particular service in the cascade. These papers were categorized as experiences of implementing a PMTCT project, [16-35]. The remaining 26 papers were related to the individual components of the PMTCT cascade.

The 20 papers on experiences of implementing a PMTCT program were further categorized as studies describing only the uptake of different services in PMTCT programs implemented in particular health facilities (N = 9), classified as a numeric summary of the uptake of services [16,19,21-23,26,27,33,34], studies dealing with understanding the barriers to accessing PMTCT services (N = 3) [18,20,35], with assessing the feasibility of expanding the PMTCT program in rural areas with the help of traditional birth attendants, classified as outreach (N = 1) [24], with evaluating the cost effectiveness of PMTCT services or programs (N = 6) [25,28-32], and with understanding the experiences entailed in the integration of the PMTCT program with family planning and sexual and reproductive health services (N = 1) [17].

In distinguishing the different individual components in the cascade, we adopt the categorization of Ciaranello et al. (2011) which follows the experiences of mothers in the antenatal care [36]. Of the 26 papers on the individual components of the cascade, none were related to access to antenatal care (ANC), 14 were related to HIV counseling and HIV testing [37-50], 6 to provision of ARV to mother and/or baby [51-56], 4 to infant feeding [57-60], and 1 each to obstetric care for HIV-infected women [61] and the diagnosis of HIV among infants [62]. The studies on counseling and HIV testing were divided into two sub-categories. Studies that investigated women’s perspective on counseling and HIV testing were categorized as acceptability and utilization (N = 8) [38,39,42,45,46,48-50], and studies carried out from the perspective of health systems were classified as feasibility and provision of counseling and HIV testing (N = 6) [37,40,41,43,44,47].

### Description of public health literature on PMTCT

The main characteristics and summaries of the outcomes of the studies related to the provision and/or utilization of services (N = 46) are provided in Table 1. The numeric summary of uptake of services is provided in Table 2. The findings of the studies are discussed below, starting with the experiences in implementing a PMTCT program and followed by the studies on the individual components of the cascade.

**Table 1:** Summary of public health literature on PMTCT in India

Experiences of implementing a PMTCT program (N = 20)				
**Reference**	**State**	Facility*	**Objective**	**Main results**
***Numeric summary of uptake of services (N = 9)***				***Table 2***
***Barriers to accessing PMTCT services (N = 3)***				
Hancart Petitet et al. (2008) [35]	Tamil Nadu	Public	To explore sociocultural factors limiting women’s access to PMTCT services.	Lack of caregiver’s access to information, inadequate attention to social and gender issues and lack of decentralization of PMTCT activities affect access to PMTCT.
Rahangdale et al. (2010) [18]	Karnataka	Public	To study the role of stigma on access to PMTCT services.	Women who experienced stigma with health care providers, community, & family felt that it was a barrier to access services and disclosure of HIV status to avail PMTCT services was not perceived as an option.
Panditrao et al. (2011) [20]	Maharashtra	Private (N = 734&770)	To understand the socio-demographic factors associated with loss to follow-up (LTF).	10.9% women were LTF before delivery and 19.6% after delivery. Factors associated with LTF were low education, poor socioeconomic status, late registration in the program and having HIV uninfected partner.
***Outreach (taking PMTCT services beyond ANC clinics) (N = 1)***				
Madhivanan et al. (2010) [24]	Karnataka	- (N = 417)	To understand the knowledge and attitudes of traditional birth attendants (TBA) about HIV.	Only 12% TBA had heard about AIDS. Among them, knowledge about modes of transmission and PMTCT was low (44%).
***Cost effectiveness (N = 6)***				
Dandona et al. (2005) [28]	Andhra Pradesh	Public	To estimate the total and unit cost of providing voluntary counseling and testing (VCT) services.	The cost per client varied 6 fold among VCTCs (range US$ 2.92–17.14). The incremental cost of providing complete VCT services to each HIV-positive and HIV-negative client was US$ 2.54 and US$ 1.22, respectively.
Kumar et al. (2006) [25]	-	Public	To estimate the additional costs of universal HIV screening program for pregnant women.	Comparison of universal screening program vs. program restricted to high prevalence states showed that implementation of program only in high prevalence states would achieve 45% of the target in 20% cost.
Dandona et al. (2008a) [30]	Andhra Pradesh	Public	To assess the cost and efficiency of the PMTCT centers in Andhra Pradesh.	The cost per mother-neonate pair who received NVP showed a wide variation, ranging from US$ 98 to US$ 4,047. Cost was inversely related to the scale of the program.
Dandona et al. (2008b) [29]	Andhra Pradesh	Public	To estimate the changes in the unit cost of VCT and sex-workers program between 2002–2003 and 2005–2006.	Over 3 years, the unit cost of VCT dropped by half and it increased 2.4 times for services provided to sex workers due to increases in male condom distribution, staff salaries and training, and treatment for sexually transmitted infections.
Dandona et al. (2009a) [32]	Andhra Pradesh	Public	To conduct composite economic analysis of HIV prevention interventions to inform efficient utilization of resources.	The highest number of HIV infections averted per 1000 persons receiving an intervention was for MSM and women sex worker programs, followed by STI clinics and blood banks, whereas the lowest was for IEC for the general public.
Dandona et al. (2010) [31]	Andhra Pradesh	Public	To measure cost effectiveness of HIV prevention interventions by estimating disability adjusted life years (DALY) saved.	The cost per DALY saved was<US $50 for blood banks, MSM, VCT, PPTCT, STI and women sex-workers programs and>US $100 and up to US $140 for street children, condom promotion, workplace and mass media programs.
***Integration of family planning (FP)/sexual and reproductive health (SRH) services in PMTCT program (N = 1)***				
Rutenberg et al. (2005) [17]	-	-	To review field experiences with provision of family planning services within PMTCT.	High acceptance of sterilization among women suggests the program’s priority of reducing the number of children born to HIV-infected women rather than ensuring mother’s reproductive health and rights.
**Studies on individual components of the cascade (N = 26)**				
***Counseling and HIV testing (N = 14)***				
Acceptability and utilization of counseling and HIV testing by women (N = 8)				
Brown et al. (2001) [38]	Tamil Nadu & Karnataka	Public (N = 666)	To assess attitudes of pregnant women towards prenatal HIV testing and ARV prophylaxis.	86% agreed to undergo prenatal HIV testing, 21% would make this decision independently while 46% said their husband would have to decide. 97% said that they would take ARV if needed.
Shankar et al. (2003) [48]	Maharashtra	Public (N = 94 in ANC & 50 in DR)	To assess acceptability of HIV testing in ANC and delivery room (DR).	Acceptance of HIV testing was 83% in ANC clinics and 68% in the DR. Partners demonstrated very strong support for their wives to make such decisions independently.
Rogers et al. (2006) [45]	Karnataka	Private (N = 202)	To assess knowledge about VCT among pregnant women in rural area.	85% of women were willing to be tested. 94% had heard of HIV/AIDS and 60% had good knowledge about modes of transmission. However, 48% did not know about PMTCT.
Samuel et al. (2007) [46]	Tamil Nadu	Public (N = 3722)	To assess acceptance of educational session and VCT by pregnant women and to study HIV seroprevalence.	3,691 (99.2%) agreed to participate in the group educational session and 3,715 (99.8%) had VCT. Baseline knowledge regarding HIV was limited and a highly significant improvement was observed (*P*<0.0001) in the post-educational session.
Sinha et al. (2008) [49]	Maharashtra	- (N = 400)	To assess HIV testing utilization among rural women.	Recently pregnant rural women report low HIV testing (3.3%).
Vajpeyee et al. (2009) [50]	Not specified	- (N = 1169 men, 581 women)	To report VCT experience at a tertiary care center	Of 1750 participants, 67 percent were men. The main reason for seeking VCT was a history of sexual risk behavior (54.7%) for men and HIV-positive status of their spouse (38.7%) for women. Perceived discrimination based on serostatus was very high.
Dandona et al. (2009b) [39]	Andhra Pradesh	- (N = 12994)	To assess the uptake of HIV testing and study reasons for undergoing HIV testing.	The uptake of HIV test was higher in women (27.2%) than in men (18.8%). Increasing education level, urban area residence and having a job were significantly associated with the uptake. Pregnancy (57.4%) was the most common reason for seeking HIV test for women.
Kandwal et al. (2010) [42]	Andhra Pradesh	Public	To study effect of stigma on women’s access to HIV testing centers.	Geo-spacial analysis suggested that women go to a different *mandal* (sub-district) to be tested for HIV because of stigma.
Feasibility and provision of counseling and HIV testing (N = 6)				
Bharucha et al. (2005) [37]	Maharashtra	Public (N = 6702)	To examine the eligibility and acceptability of VCT among pregnant women in labor	49% of the eligible women could be provided VCT in labor room (LR). VCT in the LR was feasible but it would be of greater value where there is little or no ANC care available.
Pai et al. (2008) [44]	Maharashtra	Private (N = 1252)	To investigate the impact of round-the-clock, rapid HIV testing in rural India.	98% of the women accepted HIV testing. A program of round-the-clock rapid HIV testing, including pre-partum and extended postpartum counseling sessions was shown to be feasible.
Sastry et al. (2004) [47]	Maharashtra	Public (N = 224)	To develop tools to enhance, standardize and improve the communication of messages during the group education and counseling sessions.	Visual aids during group counseling sessions increased women's overall understanding of key issues regarding informed consent from 38% to 72%. If these same visuals were reinforced during individual counseling, improvements in women’s overall comprehension rose to 96%.
Gupta et al. (2007) [41]	Tamil Nadu	Public (N = 213)	To assess effectiveness of group perinatal counseling on HIV knowledge.	The knowledge scores after group pre-test counseling increased by 21%. The study showed that group counseling sessions achieved small gains in HIV knowledge.
Dhadwal et al. (2009) [40]	Haryana & Himachal Pradesh	- (N = 82)	To assess the impact of 12-day PPTCT counselor training program on improving counseling skills.	There was significant improvement in the post-test scores after counselor training. The average gain index ranged from 33% to 37% for the three batches.
Orne-Gliemann et al. (2010) [43]	Indian site: Maharashtra	Private	To understand the acceptability and feasibility of couple-oriented prenatal counseling (COC) among women in ANC clinics.	COC was considered by the respondents to be a feasible and acceptable strategy to actively encourage men to participate in prenatal HIV counseling and testing and overall reproductive health services.
***ARV assessment/provision/utilization (N = 6)***				
Samuel et al. (1996) [55]				Full paper not available
Sinha et al. (2007) [56]	Maharashtra	Public (N = 467)	To determine prevalence of anemia, (Hb<10 gm/Dl) and assess Zidovudine (ZDV) use and toxicity in HIV-positive pregnant women.	Prevalence of anemia was 38.7% and ZDV use was not associated with persistent or worsening anemia. At delivery, regardless of anemia status at enrollment, women receiving ≥ 2 weeks of ZDV were 70% less likely to be anemic compared with women who were not prescribed ZDV.
Read et al. (2007) [54]	Tamil Nadu	Public (N = 60)	To assess safety of Zidovudine and Nevirapine (NVP)	Long-term ZDV (with or without concomitant NVP prophylaxis) was well accepted and well tolerated.
Mawar et al. (2007) [51]	Maharashtra	-	To understand concerns and experiences of women regarding PMTCT.	HIV-infected pregnant women perceived PMTCT through AZT as a useful means of enabling them to fulfill their social responsibility of bearing a child, free from HIV infection.
Patel et al. (2009) [53]	Gujarat	Private (N = 88)	To evaluate efficacy of 3-drug combination ART for PMTCT.	MTCT was 5.55% on intention-to-treat analysis and 1.11% on as-treated analysis. Overall effectiveness was comparable to other published studies.
Murthy et al. (2011) [52]	Maharashtra	Public (N = 73)	To assess safety of single-dose NVP to mother and baby.	Mother and child, followed for 1 week postpartum, did not show serious and adverse reaction to NVP.
***Delivery/repeat HIV testing (N = 1)***				
Mukherjee et al. (2010) [61]	Tamil Nadu	Public (N = 362)	To determine the cost effectiveness of childbirth strategies for mothers receiving NVP.	Vaginal delivery was cost-effective as compared to elective Caesarian section (CS) for women receiving NVP. The incremental cost of preventing an additional HIV transmission through CS was INR 76,000.
***Infant feeding (n = 4)***				
Phadke et al. (2003) [57]	Maharashtra	Public (N = 148)	To assess post-partum morbidity among infants receiving replacement feeding for PMTCT.	Replacement feeding, primarily diluted animal milk was associated with higher rate of hospitalization (0.093 hospitalizations per 100 person-days). 14% infants required hospitalization within the 1st 6 months of life.
Suryavanshi et al. (2003) [60]	Maharashtra	Public (N = 101)	To assess factors influencing infant feeding decisions of HIV-infected mothers.	Equal number of women (44%) intended to either breast-feed or use top feeding. Lack of disclosure, lack of funds, poor hygienic conditions and risk of social repercussions were more commonly noted as reasons to breast-feed.
Shankar et al. (2005) [59]	Maharashtra	Public (N = 240)	To examine how the infant feeding recommendations have been actualized within the local context.	There was a significant increase (47%) in the number of women intending to breast-feed from 2000–2004. Availability of local information on morbidity and the influence of counselors in making infant feeding choice were important factors for the rise.
Read et al. (2010) [58]	Tamil Nadu	Public (N = 60)	To assess the infant feeding choices of HIV-1-infected women	One-third of women did not breast-feed their infants. Of those who initiated breast-feeding, the median duration of breast-feeding was approximately 3 months.
***Infant diagnosis and linkage to postnatal care (N = 1)***				
Agrawal et al. (2008) [62]	Uttar Pradesh	Public	To report the false positive test results of DNA PCR test for HIV diagnosis in infants.	Three HIV-exposed infants who received PMTCT prophylaxis and who did not receive breastfeeding were tested positive by DNA PCR at 6 weeks of age and were tested negative by two different ELISA tests after 18 months.

*Sample size for quantitative studies is presented in parenthesis.

**Table 2:** Numeric summary of uptake of services in the PMTCT cascade (N = 9)

Reference	Facility	Location	Pretest	Women tested	Post-test	Women infected*	Mother ARV uptake	MTCT rate**
Marchant et al. (1997) [22]#	Private	Mumbai, Maharashtra	-	48579	-	1.5%	9%	0% (N = 30)
Marchant et al. (2001) [23]#	Private	Mumbai, Maharashtra	-	82590	-	1.41%	38%	5.8% (N = 68)
Nagdeo et al. (2007) [34]	Private	Nagpur, Maharashtra	7897	90%	-	0.72%	39%	Not stated
Sinha et al. (2008) [16]	Public	High prevalence states in India	179681	79%	-	0.41%	63%	Not stated
Dash et al. (2009) [27]	Public	Berhampur, Orissa	7066	65%	-	1.03%	51%	Not stated
Parmeshwari et al. (2009) [19]	Public	Namakkal, Tamil Nadu	7866	100%	-	0.77%	84%	Not stated
Chaudhari et al. (2010) [33]	Private	Kolkata, West Bengal	49580	96%	95%	0.17%	43%	27.3% (N = 11)
Joshi et al. (2010) [26]	Public	Ahmadabad, Gujarat	187196	83%	74%	0.35%	55%	3.6% (N = 84)
Mandal et al. (2010) [23]	Public	Siliguri, West Bengal	23812	83%	-	0.56%	30%	29.4% (N = 34)
**Average**				**86%**		**0.77%**	**46%**	**13.2%**

# the duration for reporting the data on number of women tested is not the same for reporting the number of HIV infected women provided ARV.

*reported rates of HIV prevalence in ANC.

** reported rates of mother to child transmission.

### Studies on experiences of implementing a PMTCT program (N = 20)

The 20 papers in this category reported the uptake of various services offered under PMTCT, such as the number of women who were counseled, tested, detected as HIV-infected and who received ARV prophylaxis in a particular hospital[19,21,27], or the compilation of data from several PMTCT centers [16] (See Table 2), or were related to the implementation of the program at the macro level, for example, cost effectiveness studies [25,30,32] or at the micro level [20,35,63], for example, a study on the knowledge of traditional birth attendants [24]. Of the 18 facility-based studies, 13 used data from public health facilities, and 5 used data from private health facilities [20-22,33,34]. Of these latter five, four provided a numeric summary of the program and only one paper studied factors associated with women who were lost to follow-up in the program implemented in a private sector facility [20].

#### Numeric summary of uptake of services in the PMTCT cascade (N = 9)

The uptake of the cascade of services in PMTCT, particularly ARV uptake among mothers, was considerably low in many studies in the private [21,22,33] as well as in the public sectors [22,26] with high variability among studies. The uptake of HIV testing (N = 7) ranged from 65% to 100% (average uptake 86%) from counseling to HIV testing. The uptake of ARV (N = 9) among HIV infected women ranged from 9% to 84% (average uptake 46%). (Table 2). Similarly there was wide variation in the reported rates of MTCT (N = 5) in these studies ranging from 0% to 29%. Most studies summarizing the uptake of services used single dose NVP as ARV protocol except for two studies by Marchant et al. in 1997 [22] and 2001 [21] that reported uptake of AZT among women attending a private health facility in Mumbai. However, ARV protocol was not specified in one study [16]. While information on the number of women provided with pre-test counseling and HIV testing is given, information lacked on the number of women that received post-test counseling in most of these studies. The average ANC prevalence (N = 9) was 0.77 (range 0.17 to 1.5).

#### Barriers to accessing PMTCT services (N = 3)

Only three published studies, two in the public and one in the private sector, have looked at the barriers to accessing PMTCT services. The barriers identified in these studies were a lack of training among health care providers and inadequate attention to social and gender issues [35], perceived stigma and experiences of discrimination in health facilities [18], and other demographic and economic factors [20], such as poor education, low economic status of women and lack of support from partners especially to women who have HIV un-infected partners.

#### Outreach (taking PMTCT services beyond ANC clinics) (N = 1)

While most studies related to implementation of PMTCT program focused on services delivered in the health facilities, there was only one study by Madhivanan et al. (2010) [24] that looked at the role of traditional birth attendants (TBA) in implementing PMTCT project in community setting. The study reported that majority of TBA lack basic knowledge about HIV/AIDS and safe delivery practices and recommended further research to examine whether TBA should be trained and integrated into PMTCT program.

#### Cost effectiveness (N = 6)

Among the studies on cost effectiveness, two studies reported the cost effectiveness specifically of the PMTCT program [25,30] whereas the other four studies - all from Dandona et al. - were related to Voluntary Counseling and Testing (VCT) services and HIV prevention interventions which included PMTCT as one of the components. Among the studies focusing on PMTCT, Kumar et al. (2006) compared cost effectiveness of universal screening program against the screening program restricted only to high HIV prevalent states and reported that the later strategy would be more cost effective. The study by Dandona et al. (2008a) - based on the analysis of 16 PPTCT centers - observed significant variation in these centers in the economic cost per mother-neonate pair who received Nevirapine. The average economic cost per post-HIV-test counseled pregnant woman was Indian Rupees (INR) 98.9 (US$ 2.23) [ranging 2.7-fold from INR 71.4 (US$ 1.61) to INR 189.9 (US$ 4.29)] and average economic cost per mother-neonate pair who received Nevirapine to be INR 10,210 (US$ 231) [ranging 41-fold for the 16 centers from INR 4,354 (US$ 98) to INR 179,175 (US$ 4,047)]. This variation within centers was observed to be significantly related to HIV prevalence at these centers. Very high unit cost at some centers was inversely related to HIV prevalence among pregnant women. Overall the studies on cost effectiveness, mostly carried out from the government’s perspective, indicated that large-scale programs that are integrated with other services would be more cost effective.

#### Integration of family planning (FP)/sexual and reproductive health (SRH) services in PMTCT program (N = 1)

Except for the paper by Rutenberg et al. (2005), there were no other papers on the integration of the PMTCT program with other services. This multi-country study reported the field experiences of integrating family planning services into PMTCT programs [17] and highlighted that the priority of PMTCT programs seems to be just to reduce the number of HIV infected children born to HIV-infected women, rather than ensuring HIV-infected women’s reproductive rights [17] because of a lack of focus on their reproductive choices.

### Studies on individual components of the cascade (N = 26)

#### Access to antenatal care (ANC) (N = 0)

The first component of the cascade of PMTCT is women’s access to antenatal care (ANC) services in the health facilities so that they can be offered counseling and HIV testing. In the current review no published study was identified that focused on women’s access to ANC care in India.

#### Counseling and HIV testing (N = 14)

The antenatal counseling and HIV testing of pregnant women have been studied in terms of (a) women’s acceptance and utilization of counseling and testing, and (b) the feasibility and provision of counseling and HIV testing. A high degree of acceptability of counseling and HIV testing during pregnancy and labor has been documented [15,37,38,45,46,48]. The need to involve the husband for decision making regarding HIV testing during pregnancy varied [38,48]. Most of these studies were conducted at public health care facilities. A community-based study [49] from rural Maharashtra reported very low utilization of HIV testing among rural Indian women of child- bearing age. This study found that a lack of discussion by antenatal care providers with women and a lack of awareness of existing testing services among women were major barriers to HIV testing during pregnancy. Another community-based study from Andhra Pradesh which assessed the distribution of HIV testing in a population reported a high proportion of HIV testing in the private sector. The uptake of HIV testing was higher in women (27.2%) than in men (18.8%), with pregnancy (57.4%) being the most common reason for undergoing the test [39]. A recent study from Andhra Pradesh demonstrated the role of stigma in reducing the utilization of testing facilities and, based on the geospatial analysis it reported that women go to a different mandal (sub-district) to be tested for HIV [42].

Studies related to the provision of counseling and HIV testing looked at the characteristics of health systems, such as the logistics of providing counseling and HIV testing in the labor room, the content and process of counseling, and ways to involve men in antenatal care. Two studies conducted at tertiary care hospitals reported that it is feasible to conduct rapid HIV testing during labor [37,44]; one of the studies adopted a brief prepartum and extended postpartum counseling session [44]. These studies inferred that while it is challenging to provide voluntary counseling and HIV testing (VCT) to all women in the delivery room, it could be an important venue for testing otherwise low-risk women. The availability of testing in the labor room would be of great benefit where there is little or no ANC care available. Studies regarding the process and content of counseling suggest that group antenatal counseling sessions have little impact on women’s understanding of modes of HIV prevention and on informed written consent for HIV testing [41,47]. A study from Pune [47] reported that the inclusion of culturally appropriate visual aid to group education and counseling substantially improved pregnant women’s understanding about the key issues related to informed consent for HIV testing (from 38% to 72% in group counseling and 96% in individual counseling).

There is a scarcity of literature on the strategies to involve men, particularly in the context of PMTCT. The only available study from the feasibility phase of a multi-country trial (ANRS-12127, Prenahtest), investigating the feasibility of enhanced prenatal counseling in improving men’s involvement in ANC in the context of PMTCT, reported that couple-oriented counseling was considered by health care providers and pregnant women to be a feasible and acceptable strategy to actively encourage men to participate in prenatal counseling and HIV testing and overall reproductive health services [43].

#### ARV (assessment/provision/utilization) (N = 6)

Except for one study by Mawar et al. (2007), all other studies in this category were related to the clinical aspect of providing ARV to mother and baby, such as safety and toxicity of ARVs (Zidovudine and Nevirapine) and the prevalence of anemia due to Zidovudine use. Zidovudine (AZT) and Nevirapine were reported to be safe and it was suggested that mild anemia should not limit use of Zidovudine in India [56]. The qualitative study by Mawar et al. (2007) reported that the availability of an ‘easily accessible’ and ‘low cost’ intervention (AZT), which otherwise would have been prohibitively expensive, is a major factor influencing HIV infected women’s decision to continue their pregnancy. The study also emphasized that health care providers require training in order to focus on women-centered approaches as the program scales up [51].

In the reviewed literature there is no study related to immunological assessment of pregnant HIV infected women by CD4 testing to determine their eligibility to receive combination ARV or HAART.

#### Delivery/repeat HIV testing (N = 1)

The only available study which looked at the cost effectiveness of childbirth strategies for HIV-infected pregnant women receiving Nevirapine was carried out by Mukherjee et al. (2010). The study found that vaginal delivery is not only cheaper but also cost-effective, as compared to elective Caesarian section (CS). In view of the economic benefits, the study supports the policy of normal vaginal delivery for HIV-infected women receiving Nevirapine [61].

There is no study from India investigating the feasibility and cost effectiveness of repeat HIV testing among pregnant women in third trimester of their pregnancy to detect women who get sero-converted during pregnancy and can be offered PMTCT interventions.

#### Infant feeding (N = 4)

Of the four papers, three were based on data from a single urban tertiary hospital in Pune, Maharashtra, and the fourth on data from two public hospitals in rural Tamil Nadu in South India.

Studies on infant feeding in India have investigated the morbidity and mortality associated with replacement feeding as well as infant feeding decision making and practices of HIV-infected mothers. A study from Pune by Phadke et al. (2003) [57] documented a substantial and highly significant (p<0.0001) increase in hospitalization of replacement-fed infants compared with their breast-fed counterparts; several limitations were mentioned regarding the interpretation of the data. Despite this, the results supported the recommendation that each community needs to assess the relative risks and benefits of their own available feeding options for infants born to HIV-infected mothers. The study also underlined the need for new interventions that make exclusive breast-feeding safer for both mother and infant, in settings such as India, where access to affordable and safe infant formula is limited and where social stigmatization of non-breast-feeding mothers is a concern. Another study from Pune by Suryavanshi et al. (2003) [60] looked at the factors that influence the infant feeding decisions of HIV-infected mothers. The study reported that the hospital counselor had an important role in assisting women in their intended feeding choice as well as in actual practice. The study also emphasized the need for future research to address the nutritional value and safety of milk used in top feeding.

Other studies from Pune [14,59] and Tamil Nadu [58] documented the complexities inherent in the decision making regarding infant feeding and the difficulty of adhering to exclusive breast-feeding. In the study by Read et al. (2010), one-third of the mothers initiated breast-feeding and the median duration of breast-feeding was approximately 3 months. The proportion of exclusive breast-feeding declined from approximately 70% during the first week of life to 0% by the time of the 8th month visit. The paper by Shankar et al. (2005) discussed how global recommendations on infant feeding are translated into practices and highlighted the need for tailoring policy recommendations by using local information [59]. The authors also suggest the need for developing a rapid decision-making algorithm which would allow identification of the healthiest feeding choice and minimize the pitfalls of promoting homogeneous practices lacking site-specific, evidence-based evaluation.

#### Infant diagnosis and linkage to postnatal care (N = 1)

The only published study related to the diagnosis of HIV among exposed children is a letter to the editor by Agrawal et al. (2008) [62] reporting false positive results of DNA PCR tests conducted for the diagnosis of HIV among infants. No studies exist on linking HIV infected women and children to postnatal care in India.

## Discussion

In the literature, empirical work on PMTCT in India (N = 134) seems to be dominated by epidemiological studies (N = 60). The 46 papers related to the utilization/provision of the cascade of PMTCT services were mostly from the four high prevalence states in southern India and from the public sector. Of these 46 papers, 20 were related to experiences of implementing a PMTCT program and 26 were related to individual components of the cascade of PMTCT services.

Studies related to experiences of the implementation of PMTCT programs have been largely restricted to providing a numeric summary of the program with some efforts to understand the cost effectiveness of the programs and barriers in providing PMTCT services particularly counseling and HIV testing. The current literature shows high rates of drop out especially among HIV infected women before they could receive ARVs. Studies also suggest that the cost effectiveness of PMTCT programs can be improved if they are integrated with other services and if PMTCT services can demonstrate other health benefits than just preventing HIV transmission. Lack of knowledge about the availability of HIV testing facilities among women and fear of stigmatization from health providers seem the most important barriers in accessing counseling and HIV testing services. High rate of testing in private health facilities - where PMTCT program are not implemented - also suggest the possibility of uninformed testing of pregnant women in these facilities. However, there is a lack of literature on coverage of PMTCT services in private health system. Research is also needed on systematic program evaluation documenting potential health benefits of ANC counseling to the mother and the child and assessing the outcome of PMTCT program by measuring outcome indicators such as HIV free survival among children and linking HIV infected women and children to care. Literature on best practices in the field setting that are shown to improve uptake of PMTCT services is lacking as well.

Our review on the individual components of the PMTCT cascade showed that there is a lack of literature on factors associated with pregnant women’s access to ANC care, HIV infected women’s immunological assessment (using CD4 testing) for evaluating their eligibility to receive combination ART or HAART, feasibility and cost effectiveness of repeat HIV testing among pregnant women who are in third trimester of their pregnancy, feasibility and uptake of early infant diagnosis and factors related to linking HIV infected women and children to postnatal care. Studies on HIV counseling and testing of pregnant women suggest that it is acceptable to women and feasible to provide in both public and private health facilities. The studies on infant feeding show high morbidity among non breast-fed infants. However, high proportion of HIV infected women in these studies had opted for replacement feeding (mainly animal milk). Among women who chose to breast-feed, practicing exclusive breastfeeding was observed to be challenging.

Except for the papers providing numeric summary of the uptake of PMTCT services, the overall quality of the papers was good. However, generalizability of the findings particularly for studies related to cost effectiveness that are restricted to a single state and studies on infant feeding that are carried out in a single tertiary care hospital might be low. The studies on the numeric summary of uptake of services did not provide data on all the components of the cascade of PMTCT services and the sample sizes for some studies were low [19,27,34], which made it impossible to summarize the percentage drop-outs at each step of the cascade.

The rates of uptake of PMTCT services and the findings of cost effectiveness of programs varied across studies. While the variations in the cost across studies on cost effectiveness could be because of the comparison of different programs and services in these studies, the variations in rates of uptake could be because of the different program strategies or due to lack of consistency in the methodology for estimating the uptake rates. For example, in the study by Joshi et al. (2010) the reported ARV uptake was 92%. However, this high rate was mainly because the data of only the women who delivered in the hospital were considered for estimating the ARV uptake among mother baby pair leaving out 36% of the HIV infected women who were lost to follow up. The high rate of uptake in the two studies by Parameshwari et al. (2009) and Panditrao et al. (2011) (reported under barriers in accessing care) - both part of the Elizabeth Glaser Pediatric AIDS Foundations’ PMTCT program in India - might be because of the program implementation strategies, such as quality of counseling, educational material used, monitoring and evaluation systems and demographic and sociocultural factors [20] of women enrolled in these programs. Similar to most papers describing programmatic experiences the paper by Parameshwari et al. (2009) was based on a descriptive analysis of a small number of HIV infected women (N = 56) whereas Panditrao et al. (2011) analyzed program data from 2002 to 2008 of 734 HIV infected women who were enrolled during pregnancy and 770 HIV infected women who reported a live birth in order to understand factors associated with loss to follow-up (LTF) before delivery and after delivery respectively using multivariate statistical techniques. Most of the current literature on program experiences does not describe the program implementation strategies which limit analysis on their association with the uptake. There is an urgent need for a systematic understanding of these factors in India and to improve the reporting of existing program data to identify strategies and practices that could improve efficacy of PMTCT program.

In this review, data about the program from public as well as private health facilities suggest that a substantial proportion of HIV infected women enrolled in a PMTCT project drop out, without receiving the complete cascade of services. Loss to follow-up of women enrolled in PMTCT programs has been recognized as a major factor affecting the efficacy of the program in resource-poor countries [64,65]. While there have been some studies from Africa on risk factors relating to loss to follow-up, a systematic understanding of the factors affecting continued access of services among HIV-infected Indian women is lacking [20].

Although this review on PMTCT literature in India has uncovered only limited evidence regarding factors affecting the uptake of counseling and HIV testing among pregnant women - such as the availability of a HIV testing facility, discussion about HIV testing with care providers and perceived stigma from the health facilities -, elsewhere there is burgeoning literature on the factors affecting such uptake as well as the integration of PMTCT programs with other health services. A systematic review of factors affecting counseling and HIV testing, mainly from Africa, suggests that provider-initiated counseling and testing, group pre-test counseling sessions and rapid HIV testing with same-day results increase the uptake of counseling and testing [66]. However, another systematic review of South African studies suggests that in addition to these factors quality of counseling and contextual factors such as HIV prevalence, human and physical resources significantly affect counseling, and the review recommends that best available evidence from research studies regarding these factors should be used to develop guidelines for the local context [67]. While a family-centered approach in implementing PMTCT and integration of the PMTCT program with other services has been generally recommended in many countries including India [17,68], a recent review by Cochrane on the efficacy of integrating PMTCT with other services argued that evidence from resource-poor countries does not allow one to conclude that integrated care is more effective than non-integrated or partially integrated care. The influence of the socio-cultural context in the uptake of services highlighted in these studies strongly suggests the need to conduct research locally to determine the factors affecting the efficiency of PMTCT services and the benefits of integrating PMTCT with other services [69].

This review has shown that evidence on experiences in providing PMTCT services in the private health care system is limited as compared to public health care system. This is striking as, in India, the private health care system plays a significant role in the delivery of health care. Approximately 70 percent of urban households and 63 percent of rural households access care from the private health sector [70]. Though the role of the private sector is recognized in the third National AIDS Control Program (NACP-3) [71], efforts to include it have been sporadic. There is a complete lack of systematic knowledge on the current coverage of PMTCT in the private sector and on the referral mechanisms for women detected as HIV-infected in the private sector. There is an urgent need for assessing the most effective models for public-private partnerships (PPPs) in the country.

The published public health literature also seems to be geographically skewed and dominated by the analysis of data from a few centers. Of the 42 studies using data at the district/state level, 16 papers were from Maharashtra (of these, eight were from a single tertiary care center in Pune), eight from Tamil Nadu, seven from Andhra Pradesh (out of which five were cost effectiveness studies) and four were from Karnataka. It is important to note that in spite of being one of the high prevalence states since the beginning of AIDS epidemic in India, nothing has been published from Manipur and Nagaland. The composition and performance of health systems in these two north-eastern states differ from the other four high HIV prevalence states in the country. As the provision and utilization of PMTCT services depends on sociocultural and health-system-related factors, understanding of these factors across states would be essential for effective implementation of the program in the entire country. It is also interesting to note that except one all the papers on cost effectiveness are authored by Dandona et al. Cost effectiveness analysis is one of the important tools for program planning and implementation. Therefore allowing a richer academic discourse on this issue by attracting and training more scholars in this field might be beneficial.

## Conclusions

In order to provide universal access to PMTCT services and to virtually eliminate pediatric HIV in India there is need to a) increase availability of counseling and HIV testing facilities b) increase accessibility of these facilities by addressing the issues of discrimination in health facilities through training and sensitization of healthcare providers and c) increase awareness among women about availability of such facilities. Considering the overall low HIV prevalence in the country, integrated PMTCT programs that can demonstrate other health benefits to the mother and the child would be more cost effective. On a programmatic level there is an urgent need to evaluate alternative models of implementation of PMTCT programs in order to maximize the reach of PMTCT services to HIV infected women in least cost. Strengthening the linkages between public and private facilities could maximize the reach of PMTCT services to infected women. However, currently the knowledge on effective models of public private partnerships for PMTCT is lacking.

The current national PMTCT program faces double jeopardy due to high rates of drop out of women in the PMTCT cascade and continued use of least efficacious single dose Nevirapine as the PMTCT protocol. Systematic exploration of factors affecting women’s continued access to PMTCT services is needed along with rapid rollout of more effective PMTCT protocol recommended by the WHO to increase efficacy of the program. The systematic review also highlights the inadequacy of proper analysis and representation of program data which could be an important source of experiential knowledge helpful in program implementation.

The importance of well-planned health research has been recognized as fundamental to the improvement of health in all countries [72]. However, it has been shown that public health research output from India has in general been considerably low [73,74]. The findings of this systematic review also suggest that literature on the public health aspects of the PMTCT program is sparse. For a program such as the Indian PMTCT program that operates on a large scale, a comprehensive understanding of the context-driven factors affecting its efficiency is necessary. For effective planning and implementation of PMTCT programs in India, there is a need to generate systematic and more focused public health research output, including the issues related to the performance of complex health systems, geographic differences, and sociocultural factors affecting women’s access to PMTCT services. Efforts should be made to analyze and publish program-related data that facilitate the planning of research that is relevant and embedded in the local context.

## Competing interests

The authors declare that they have no competing interests

## Authors’ contributions

SD, MP, RP, VK and SK conceptualized the study. SD, MP and RP retrieved the data. SD, RP and SK participated in the analysis. SD conducted the analysis and took the lead in writing the manuscript. FJ supervised the analysis and manuscript writing. All authors helped to draft the manuscript and read and approved the final manuscript.

## Pre-publication history

The pre-publication history for this paper can be accessed here:

http://www.biomedcentral.com/1471-2458/12/320/prepub
